# Penta­potassium europium(III) dilithium deca­fluoride, K_5_EuLi_2_F_10_

**DOI:** 10.1107/S1600536809044055

**Published:** 2009-10-28

**Authors:** Anna Gagor

**Affiliations:** a W. Trzebiatowski Institute of Low Temperature and Structure Research, Polish Academy of Sciences, Okólna str. 2, PO Box 1410, 50-950 Wrocław, Poland

## Abstract

The title compound, K_5_EuLi_2_F_10_, belongs to so-called self-activated materials containing lanthanoid ions within the matrix. A common feature of these systems is a large separation between the closest lanthanoid ions, which is one of the crucial factors governing the self-quenching of luminescence. The crystal structure of K_5_EuLi_2_F_10_ is isotypic with other K_5_ *RE*Li_2_F_10_ compounds (*RE* = Nd, Pr). As expected from the lanthanoid contraction, the unit-cell volume for crystal with Eu^3+^ ions is the smallest of the three structures. Accordingly, the corresponding inter­atomic *RE*—*RE* distances are shorter. In the structure, distorted EuF_8_ dodeca­hedra and two different LiF_4_ tetra­hedra, all with *m* symmetry, are present, forming sheets parallel to (100). The isolated EuF_8_ dodeca­hedra exhibit a mean Eu—F distance of 2.356 Å. The K^+^ cations are located within and between the sheets, leading to highly irregular KF_*x*_ polyhedra (*x* = 8–9) around the alkali metal cations.

## Related literature

The structure of the isotypic Nd analogue was reported by Hong & McCollum (1979 ▶); for the structure of the Pr analogue, see: Gagor (2009 ▶). For background to bond-valence calculations, see: Brown (1992 ▶, 2002 ▶); Mattausch *et al.* (1991 ▶). Synthetic details were described by Ryba-Romanowski *et al.* (2007 ▶).

## Experimental

### 

#### Crystal data

K_5_EuLi_2_F_10_
*M* *_r_* = 551.35
Orthorhombic, 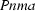

*a* = 20.5539 (6) Å
*b* = 7.7356 (2) Å
*c* = 6.8721 (2) Å
*V* = 1092.64 (5) Å^3^
*Z* = 4
Mo *K*α radiation
μ = 7.75 mm^−1^
*T* = 295 K
0.35 × 0.20 × 0.15 mm

#### Data collection

Kuma KM-4 with CCD area-detector diffractometer
Absorption correction: multi-scan (*CrysAlis RED*; Oxford Diffraction, 2007 ▶) *T*_min_ = 0.146, *T*_max_ = 0.310
23852 measured reflections
4895 independent reflections
3564 reflections with *I* > 2σ(*I*)
*R*_int_ = 0.034

#### Refinement

*R*[*F* ^2^ > 2σ(*F* ^2^)] = 0.021
*wR*(*F* ^2^) = 0.039
*S* = 0.83
4895 reflections
98 parameters
Δρ_max_ = 2.35 e Å^−3^
Δρ_min_ = −3.02 e Å^−3^

### 

Data collection: *CrysAlis CCD* (Oxford Diffraction, 2007 ▶); cell refinement: *CrysAlis RED* (Oxford Diffraction, 2007 ▶); data reduction: *CrysAlis RED*; program(s) used to solve structure: *SHELXS97* (Sheldrick, 2008 ▶); program(s) used to refine structure: *SHELXL97* (Sheldrick, 2008 ▶); molecular graphics: *DIAMOND* (Brandenburg & Putz, 2006 ▶); software used to prepare material for publication: *SHELXL97*.

## Supplementary Material

Crystal structure: contains datablocks I, global. DOI: 10.1107/S1600536809044055/wm2269sup1.cif

Structure factors: contains datablocks I. DOI: 10.1107/S1600536809044055/wm2269Isup2.hkl

Additional supplementary materials:  crystallographic information; 3D view; checkCIF report

## Figures and Tables

**Table 1:** Selected bond lengths (Å)

Eu1—F5	2.2923 (11)
Eu1—F2	2.3236 (11)
Eu1—F3^i^	2.3262 (7)
Eu1—F1	2.3918 (10)
Eu1—F4	2.3954 (7)
Eu1—F8^ii^	2.3996 (10)
Li1—F6^iii^	1.804 (3)
Li1—F1^iv^	1.862 (3)
Li1—F3^v^	1.8669 (16)
Li2—F7	1.801 (4)
Li2—F4^iii^	1.817 (2)
Li2—F8	1.844 (3)

Symmetry codes: (i) 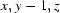; (ii) 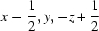; (iii) 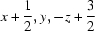; (iv) 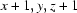; (v) 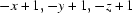.

**Fig. 1.**
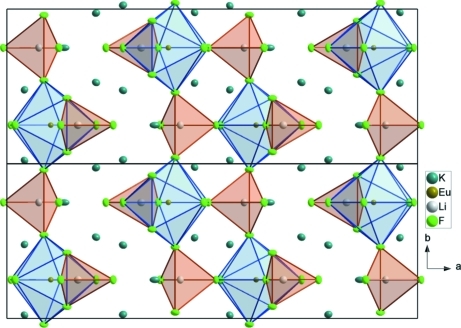
Crystal packing in K5EuLi2F10 as seen down the c axis. The thermal ellipsoids have been drawn at the 50% probability level.

## supplementary crystallographic information

### Comment

Two different LiF_4_ tetrahedra together with EuF_8_ dodecahedra form sheets expanding perpendicular to [100]. Fig. 1 illustrates the crystal packing of K_5_EuLi_2_F_10_ as seen down [001]. K1 atoms occupy cavities within the sheets and are surrounded by 9 F^-^ ions in a mean distance of 2.792 Å. Remaining potassium atoms are located between the sheets, leading to KF_9_ and KF_8_ polyhedra. The valence sums of K atoms are close to the formal charge of +1, with a slight tendency to over-bonding. The K2 ion in the K_5_EuLi_2_F_10_ structure is slightly under-bonded (S = 0.977 v.u.), whereas the Li position is over-bonded, with a 15.6% higher bond-valence sum than those expected from the formal charge of +1.

Each EuF_8_ dodecahedron is surrounded by twelve others with a shortest and longest Eu—Eu distance of 6.6968 (2) and 7.8353 (2) Å, respectively. The mean distance of Eu—Eu is 7.309 Å, with individual distances of 2× 6.6968 (2), 2× 6.8805 (2), 2× 6.8721 (2), 2× 7.7356 (2) and 4× 7.8353 (2) Å. The bond valence sums of all metal atoms have been calculated from the received structure model on the basis of the bond-valence method (Brown, 1992, 2002; Mattausch *et al.*, 1991)]: Eu 2.81, K1 1.06, K2 1.00, K3 1.09 and Li 1.16 v.u. The Eu ion is slightly under-bonded. The lower value of Eu valence may be associated with the distorted surrounding of this cation. When such distortions occur, the equal-valence rule is not strictly obeyed (Brown, 1992).

### Experimental

Preparation details were taken from Ryba-Romanowski *et al.* (2007). The K_5_EuLi_2_F_10_ crystal was grown from commercially available KF, EuF3 and LiF (Aldrich 99.99%, anhydrous) using the Bridgman method. The reagents were heated at 923 K (melting point 813 K) in a graphite crucible under argon atmosphere. The pulling rate was 1mm/h, the temperature gradient 100 %/cm.

### Refinement

In the final Fourier map, the highest peak is 0.60 Å from atom Eu1 and the deepest hole is 0.63 Å from the same atom.

### Crystal data

**Table:**

K_5_EuLi_2_F_10_	*F*(000) = 1016
*M**_r_* = 551.35	*D*_x_ = 3.352 Mg m^−^^3^
Orthorhombic, *P**n**m**a*	Mo *K*α radiation, λ = 0.71073 Å
Hall symbol: -P 2ac 2n	Cell parameters from 12717 reflections
*a* = 20.5539 (6) Å	θ = 2.8–47.0°
*b* = 7.7356 (2) Å	µ = 7.75 mm^−^^1^
*c* = 6.8721 (2) Å	*T* = 295 K
*V* = 1092.64 (5) Å^3^	Rectangular prism, colorless
*Z* = 4	0.35 × 0.20 × 0.15 mm

### Data collection

**Table:**

Kuma KM-4 with CCD area-detector diffractometer	4895 independent reflections
Radiation source: fine-focus sealed tube	3564 reflections with *I* > 2σ(*I*)
graphite	*R*_int_ = 0.034
Detector resolution: 1024x1024 with blocks 2x2, 33.133pixel/mm pixels mm^-1^	θ_max_ = 47.2°, θ_min_ = 3.1°
ω scans	*h* = −42→27
Absorption correction: multi-scan (*CrysAlis RED*; Oxford Diffraction, 2007)	*k* = −11→15
*T*_min_ = 0.146, *T*_max_ = 0.310	*l* = −14→9
23852 measured reflections	

### Refinement

**Table:**

Refinement on *F*^2^	Primary atom site location: structure-invariant direct methods
Least-squares matrix: full	Secondary atom site location: difference Fourier map
*R*[*F*^2^ > 2σ(*F*^2^)] = 0.021	*w* = 1/[σ^2^(*F*_o_^2^) + (0.02*P*)^2^] where *P* = (*F*_o_^2^ + 2*F*_c_^2^)/3
*wR*(*F*^2^) = 0.039	(Δ/σ)_max_ = 0.001
*S* = 0.83	Δρ_max_ = 2.35 e Å^−^^3^
4895 reflections	Δρ_min_ = −3.02 e Å^−^^3^
98 parameters	Extinction correction: *SHELXL97* (Sheldrick, 2008), Fc^*^=kFc[1+0.001xFc^2^λ^3^/sin(2θ)]^-1/4^
0 restraints	Extinction coefficient: 0.0234 (3)

### Special details

**Table:**

Geometry. All esds (except the esd in the dihedral angle between two l.s. planes) are estimated using the full covariance matrix. The cell esds are taken into account individually in the estimation of esds in distances, angles and torsion angles; correlations between esds in cell parameters are only used when they are defined by crystal symmetry. An approximate (isotropic) treatment of cell esds is used for estimating esds involving l.s. planes.
Refinement. Refinement of *F*^2^ against ALL reflections. The weighted *R*-factor *wR* and goodness of fit *S* are based on *F*^2^, conventional *R*-factors *R* are based on *F*, with *F* set to zero for negative *F*^2^. The threshold expression of *F*^2^ > σ(*F*^2^) is used only for calculating *R*-factors(gt) *etc*. and is not relevant to the choice of reflections for refinement. *R*-factors based on *F*^2^ are statistically about twice as large as those based on *F*, and *R*- factors based on ALL data will be even larger.To eliminate the weak reflections measured at high theta angles a 2theta limit was applied during structure refinement. The refinement on the whole data set (2theta = 47°) only slightly improved the standard deviations. Concluding, it was decided to refine the structure using a maximum measured 2theta limit. For completeness calculations the 2theta threshold was set to 28.

### Fractional atomic coordinates and isotropic or equivalent isotropic displacement parameters (Å^2^)

**Table:**

	*x*	*y*	*z*	*U*_iso_*/*U*_eq_	
K1	0.457104 (13)	0.97833 (4)	0.25084 (4)	0.01525 (4)	
K2	0.282845 (14)	0.02578 (4)	0.42686 (4)	0.01675 (5)	
K3	0.36036 (2)	0.2500	0.93720 (6)	0.01762 (7)	
Eu1	0.106855 (4)	0.2500	0.236787 (10)	0.00739 (2)	
Li1	0.92238 (15)	0.2500	0.9701 (4)	0.0131 (5)	
Li2	0.67290 (16)	0.2500	0.8419 (5)	0.0143 (6)	
F1	0.00915 (5)	0.2500	0.04773 (15)	0.01369 (18)	
F2	0.01991 (5)	0.2500	0.45288 (15)	0.0174 (2)	
F3	0.09032 (4)	0.96151 (9)	0.15506 (12)	0.01698 (15)	
F4	0.14639 (4)	0.07571 (10)	0.49951 (11)	0.01549 (14)	
F5	0.21739 (5)	0.2500	0.19250 (16)	0.0167 (2)	
F6	0.37353 (6)	0.2500	0.31189 (16)	0.01580 (19)	
F7	0.75888 (5)	0.2500	0.79160 (15)	0.0160 (2)	
F8	0.63085 (5)	0.2500	0.60493 (14)	0.01447 (19)	

### Atomic displacement parameters (Å^2^)

**Table:**

	*U*^11^	*U*^22^	*U*^33^	*U*^12^	*U*^13^	*U*^23^
K1	0.01483 (10)	0.01435 (10)	0.01657 (10)	−0.00139 (8)	−0.00065 (9)	−0.00117 (9)
K2	0.01782 (11)	0.01449 (11)	0.01795 (10)	0.00145 (9)	−0.00036 (9)	−0.00152 (9)
K3	0.0292 (2)	0.01074 (15)	0.01291 (14)	0.000	−0.00250 (14)	0.000
Eu1	0.00789 (3)	0.00732 (3)	0.00696 (3)	0.000	−0.00052 (3)	0.000
Li1	0.0131 (13)	0.0150 (14)	0.0112 (12)	0.000	0.0005 (11)	0.000
Li2	0.0160 (15)	0.0147 (14)	0.0121 (12)	0.000	0.0002 (11)	0.000
F1	0.0096 (4)	0.0171 (5)	0.0144 (4)	0.000	−0.0018 (4)	0.000
F2	0.0163 (5)	0.0221 (5)	0.0138 (4)	0.000	0.0025 (4)	0.000
F3	0.0219 (4)	0.0108 (3)	0.0183 (3)	−0.0001 (3)	−0.0047 (3)	−0.0029 (3)
F4	0.0222 (4)	0.0104 (3)	0.0139 (3)	−0.0007 (3)	−0.0029 (3)	−0.0009 (3)
F5	0.0106 (4)	0.0228 (5)	0.0168 (4)	0.000	−0.0014 (4)	0.000
F6	0.0150 (5)	0.0197 (5)	0.0127 (4)	0.000	−0.0028 (4)	0.000
F7	0.0129 (5)	0.0203 (5)	0.0147 (4)	0.000	0.0011 (4)	0.000
F8	0.0134 (4)	0.0205 (5)	0.0095 (4)	0.000	−0.0012 (4)	0.000

### Geometric parameters (Å, °)

**Table:**

K1—F8^i^	2.7148 (9)	Eu1—F5	2.2923 (11)
K1—F1^ii^	2.7344 (7)	Eu1—F2	2.3236 (11)
K1—F2^iii^	2.7451 (9)	Eu1—F3^xviii^	2.3262 (7)
K1—F6^iv^	2.7465 (8)	Eu1—F3^xix^	2.3262 (7)
K1—F4^iii^	2.7718 (8)	Eu1—F1	2.3918 (10)
K1—F1^v^	2.7863 (8)	Eu1—F4^xx^	2.3954 (7)
K1—F3^vi^	2.8163 (9)	Eu1—F4	2.3954 (7)
K1—F2^ii^	2.8359 (8)	Eu1—F8^xxi^	2.3996 (10)
K1—Li1^vii^	2.933 (2)	Eu1—Li2^x^	3.198 (3)
K1—F3^viii^	2.9804 (8)	Li1—F6^xvii^	1.804 (3)
K1—Li2^i^	3.266 (3)	Li1—F1^xxii^	1.862 (3)
K1—Li1^ix^	3.395 (3)	Li1—F3^i^	1.8669 (16)
K2—F7^x^	2.6446 (8)	Li1—F3^xxiii^	1.8669 (16)
K2—F6	2.6658 (10)	Li1—K1^xxiv^	2.933 (2)
K2—F5	2.7225 (9)	Li1—K1^xxv^	2.933 (2)
K2—F7^xi^	2.7460 (7)	Li1—K3^xvii^	3.076 (3)
K2—F8^xi^	2.7831 (7)	Li1—K1^xxvi^	3.395 (3)
K2—F5^xii^	2.8078 (8)	Li1—K1^xxvii^	3.395 (3)
K2—F4	2.8748 (8)	Li1—K2^xvii^	3.426 (3)
K2—Li2^xi^	2.965 (2)	Li1—K2^xxviii^	3.426 (3)
K2—F3^v^	3.0440 (9)	Li2—F7	1.801 (4)
K2—Li2^x^	3.262 (3)	Li2—F4^xvii^	1.817 (2)
K2—F4^xiii^	3.3700 (8)	Li2—F4^xxviii^	1.817 (2)
K2—Li1^x^	3.426 (3)	Li2—F8	1.844 (3)
K3—F4^xii^	2.5594 (8)	Li2—K2^xi^	2.965 (2)
K3—F4^xiv^	2.5594 (8)	Li2—K2^xxix^	2.965 (2)
K3—F6^xv^	2.5891 (11)	Li2—Eu1^xvii^	3.198 (3)
K3—F7^x^	2.6119 (11)	Li2—K2^xxviii^	3.262 (3)
K3—F3^v^	2.7320 (8)	Li2—K2^xvii^	3.262 (3)
K3—F3^xvi^	2.7320 (8)	Li2—K1^i^	3.266 (3)
K3—Li1^x^	3.076 (3)	Li2—K1^xxiii^	3.266 (3)
K3—F2^xvii^	3.3652 (12)		
			
F8^i^—K1—F1^ii^	125.15 (3)	F3^i^—Li1—F3^xxiii^	122.43 (17)
F8^i^—K1—F2^iii^	88.19 (3)	F7—Li2—F4^xvii^	114.17 (13)
F1^ii^—K1—F2^iii^	143.59 (3)	F7—Li2—F4^xxviii^	114.17 (13)
F8^i^—K1—F6^iv^	91.46 (3)	F4^xvii^—Li2—F4^xxviii^	95.80 (16)
F1^ii^—K1—F6^iv^	65.12 (3)	F7—Li2—F8	106.88 (17)
F2^iii^—K1—F6^iv^	135.55 (3)	F4^xvii^—Li2—F8	112.90 (13)
F8^i^—K1—F4^iii^	67.56 (3)	F4^xxviii^—Li2—F8	112.90 (13)
F1^ii^—K1—F4^iii^	137.02 (3)	F7—Li2—K2^xi^	65.12 (8)
F2^iii^—K1—F4^iii^	64.55 (3)	F4^xvii^—Li2—K2^xi^	86.08 (4)
F6^iv^—K1—F4^iii^	74.37 (3)	F4^xxviii^—Li2—K2^xi^	178.10 (12)
F8^i^—K1—F1^v^	59.07 (3)	F8—Li2—K2^xi^	66.01 (8)
F1^ii^—K1—F1^v^	91.096 (10)	F7—Li2—K2^xxix^	65.12 (8)
F2^iii^—K1—F1^v^	95.48 (2)	F4^xvii^—Li2—K2^xxix^	178.10 (12)
F6^iv^—K1—F1^v^	121.93 (3)	F4^xxviii^—Li2—K2^xxix^	86.08 (4)
F4^iii^—K1—F1^v^	123.48 (2)	F8—Li2—K2^xxix^	66.01 (8)
F8^i^—K1—F3^vi^	122.24 (3)	K2^xi^—Li2—K2^xxix^	92.03 (9)
F1^ii^—K1—F3^vi^	62.54 (2)	Li1^xxx^—F1—Eu1	163.75 (11)
F2^iii^—K1—F3^vi^	88.52 (3)	Li1^xxx^—F1—K1^xxxi^	76.71 (6)
F6^iv^—K1—F3^vi^	127.47 (3)	Eu1—F1—K1^xxxi^	93.07 (3)
F4^iii^—K1—F3^vi^	151.91 (2)	Li1^xxx^—F1—K1^xxxii^	76.71 (6)
F1^v^—K1—F3^vi^	63.93 (3)	Eu1—F1—K1^xxxii^	93.07 (3)
F8^i^—K1—F2^ii^	164.89 (3)	K1^xxxi^—F1—K1^xxxii^	100.45 (3)
F1^ii^—K1—F2^ii^	60.15 (3)	Li1^xxx^—F1—K1^iii^	91.64 (8)
F2^iii^—K1—F2^ii^	91.732 (11)	Eu1—F1—K1^iii^	100.88 (3)
F6^iv^—K1—F2^ii^	78.06 (3)	K1^xxxi^—F1—K1^iii^	88.904 (10)
F4^iii^—K1—F2^ii^	98.83 (3)	K1^xxxii^—F1—K1^iii^	162.80 (4)
F1^v^—K1—F2^ii^	135.88 (3)	Li1^xxx^—F1—K1^xxxiii^	91.64 (8)
F3^vi^—K1—F2^ii^	72.85 (3)	Eu1—F1—K1^xxxiii^	100.88 (3)
F8^i^—K1—F3^viii^	62.84 (2)	K1^xxxi^—F1—K1^xxxiii^	162.80 (4)
F1^ii^—K1—F3^viii^	62.36 (3)	K1^xxxii^—F1—K1^xxxiii^	88.904 (10)
F2^iii^—K1—F3^viii^	148.43 (2)	K1^iii^—F1—K1^xxxiii^	78.68 (3)
F6^iv^—K1—F3^viii^	62.20 (3)	Eu1—F2—K1^xvi^	110.14 (3)
F4^iii^—K1—F3^viii^	110.69 (2)	Eu1—F2—K1^v^	110.14 (3)
F1^v^—K1—F3^viii^	59.86 (2)	K1^xvi^—F2—K1^v^	80.09 (3)
F3^vi^—K1—F3^viii^	96.38 (2)	Eu1—F2—K1^xxxi^	91.98 (3)
F2^ii^—K1—F3^viii^	119.55 (2)	K1^xvi^—F2—K1^xxxi^	157.42 (4)
Li1^vii^—K1—F3^viii^	36.79 (4)	K1^v^—F2—K1^xxxi^	88.268 (11)
F8^i^—K1—Li2^i^	34.37 (6)	Eu1—F2—K1^xxxii^	91.98 (3)
F7^x^—K2—F6	85.43 (3)	K1^xvi^—F2—K1^xxxii^	88.268 (11)
F7^x^—K2—F5	85.58 (3)	K1^v^—F2—K1^xxxii^	157.42 (4)
F6—K2—F5	75.86 (3)	K1^xxxi^—F2—K1^xxxii^	95.64 (3)
F7^x^—K2—F7^xi^	148.354 (19)	Eu1—F2—K3^x^	153.25 (4)
F6—K2—F7^xi^	124.18 (3)	K1^xvi^—F2—K3^x^	90.02 (3)
F5—K2—F7^xi^	90.98 (2)	K1^v^—F2—K3^x^	90.02 (3)
F7^x^—K2—F8^xi^	132.75 (3)	K1^xxxi^—F2—K3^x^	70.57 (2)
F6—K2—F8^xi^	91.71 (2)	K1^xxxii^—F2—K3^x^	70.57 (2)
F5—K2—F8^xi^	139.19 (3)	Li1^i^—F3—Eu1^iv^	166.53 (9)
F7^xi^—K2—F8^xi^	63.94 (3)	Li1^i^—F3—K3^iii^	81.61 (9)
F7^x^—K2—F5^xii^	91.28 (2)	Eu1^iv^—F3—K3^iii^	110.42 (3)
F6—K2—F5^xii^	133.49 (3)	Li1^i^—F3—K1^xxi^	90.59 (10)
F5—K2—F5^xii^	150.205 (12)	Eu1^iv^—F3—K1^xxi^	92.43 (3)
F7^xi^—K2—F5^xii^	76.39 (3)	K3^iii^—F3—K1^xxi^	103.03 (3)
F8^xi^—K2—F5^xii^	57.98 (3)	Li1^i^—F3—K1^xxxiv^	70.22 (9)
F7^x^—K2—F4	66.62 (3)	Eu1^iv^—F3—K1^xxxiv^	97.07 (3)
F6—K2—F4	130.25 (2)	K3^iii^—F3—K1^xxxiv^	151.20 (3)
F5—K2—F4	62.29 (3)	K1^xxi^—F3—K1^xxxiv^	83.62 (2)
F7^xi^—K2—F4	83.95 (3)	Li1^i^—F3—K2^iii^	84.87 (10)
F8^xi^—K2—F4	137.60 (2)	Eu1^iv^—F3—K2^iii^	88.18 (3)
F5^xii^—K2—F4	89.28 (3)	K3^iii^—F3—K2^iii^	93.85 (3)
F7^x^—K2—F3^v^	76.20 (3)	K1^xxi^—F3—K2^iii^	161.70 (3)
F6—K2—F3^v^	62.15 (3)	K1^xxxiv^—F3—K2^iii^	78.16 (2)
F5—K2—F3^v^	135.02 (2)	Li2^x^—F4—Eu1	97.82 (8)
F7^xi^—K2—F3^v^	125.05 (3)	Li2^x^—F4—K3^xiii^	147.88 (8)
F8^xi^—K2—F3^v^	61.27 (2)	Eu1—F4—K3^xiii^	114.18 (3)
F5^xii^—K2—F3^v^	72.01 (3)	Li2^x^—F4—K1^v^	88.18 (11)
F4—K2—F3^v^	137.88 (2)	Eu1—F4—K1^v^	107.11 (3)
Li2^xi^—K2—F3^v^	94.67 (7)	K3^xiii^—F4—K1^v^	85.08 (2)
F7^x^—K2—F4^xiii^	150.54 (2)	Li2^x^—F4—K2	84.91 (11)
F6—K2—F4^xiii^	65.89 (2)	Eu1—F4—K2	106.00 (3)
F5—K2—F4^xiii^	81.15 (3)	K3^xiii^—F4—K2	83.77 (2)
F7^xi^—K2—F4^xiii^	58.49 (3)	K1^v^—F4—K2	146.79 (3)
F8^xi^—K2—F4^xiii^	58.53 (2)	Li2^x^—F4—K2^xii^	61.37 (8)
F5^xii^—K2—F4^xiii^	112.98 (2)	Eu1—F4—K2^xii^	159.13 (3)
F4—K2—F4^xiii^	127.14 (2)	K3^xiii^—F4—K2^xii^	86.55 (2)
Li2^xi^—K2—F4^xiii^	32.54 (5)	K1^v^—F4—K2^xii^	75.704 (19)
F3^v^—K2—F4^xiii^	94.98 (2)	K2—F4—K2^xii^	72.480 (17)
Li2^x^—K2—F4^xiii^	147.93 (5)	Eu1—F5—K2^xx^	114.28 (3)
F4^xii^—K3—F4^xiv^	159.74 (4)	Eu1—F5—K2	114.28 (3)
F4^xii^—K3—F6^xv^	80.751 (19)	K2^xx^—F5—K2	79.15 (3)
F4^xiv^—K3—F6^xv^	80.751 (19)	Eu1—F5—K2^xiii^	94.85 (3)
F4^xii^—K3—F7^x^	93.29 (2)	K2^xx^—F5—K2^xiii^	150.40 (4)
F4^xiv^—K3—F7^x^	93.29 (2)	K2—F5—K2^xiii^	84.342 (11)
F6^xv^—K3—F7^x^	133.01 (4)	Eu1—F5—K2^xxxv^	94.85 (3)
F4^xii^—K3—F3^v^	63.21 (2)	K2^xx^—F5—K2^xxxv^	84.342 (11)
F4^xiv^—K3—F3^v^	136.75 (3)	K2—F5—K2^xxxv^	150.40 (4)
F6^xv^—K3—F3^v^	131.84 (3)	K2^xiii^—F5—K2^xxxv^	98.89 (4)
F7^x^—K3—F3^v^	82.48 (3)	Li1^x^—F6—K3^xxxvi^	152.17 (12)
F4^xii^—K3—F3^xvi^	136.75 (3)	Li1^x^—F6—K2^xx^	98.23 (8)
F4^xiv^—K3—F3^xvi^	63.21 (2)	K3^xxxvi^—F6—K2^xx^	102.81 (3)
F6^xv^—K3—F3^xvi^	131.84 (3)	Li1^x^—F6—K2	98.23 (8)
F7^x^—K3—F3^xvi^	82.48 (3)	K3^xxxvi^—F6—K2	102.81 (3)
F3^v^—K3—F3^xvi^	73.58 (3)	K2^xx^—F6—K2	81.18 (4)
F4^xii^—K3—F2^xvii^	90.874 (19)	Li1^x^—F6—K1^xviii^	77.21 (7)
F4^xiv^—K3—F2^xvii^	90.874 (19)	K3^xxxvi^—F6—K1^xviii^	85.04 (3)
F6^xv^—K3—F2^xvii^	71.03 (3)	K2^xx^—F6—K1^xviii^	168.64 (4)
F7^x^—K3—F2^xvii^	155.96 (3)	K2—F6—K1^xviii^	89.126 (9)
F3^v^—K3—F2^xvii^	78.33 (3)	Li1^x^—F6—K1^xix^	77.21 (7)
F3^xvi^—K3—F2^xvii^	78.33 (3)	K3^xxxvi^—F6—K1^xix^	85.04 (3)
Li1^x^—K3—F2^xvii^	78.49 (6)	K2^xx^—F6—K1^xix^	89.126 (9)
F5—Eu1—F2	147.91 (4)	K2—F6—K1^xix^	168.64 (4)
F5—Eu1—F3^xviii^	96.48 (2)	K1^xviii^—F6—K1^xix^	99.84 (4)
F2—Eu1—F3^xviii^	92.40 (2)	Li2—F7—K3^xvii^	154.06 (11)
F5—Eu1—F3^xix^	96.48 (2)	Li2—F7—K2^xxviii^	92.42 (8)
F2—Eu1—F3^xix^	92.40 (2)	K3^xvii^—F7—K2^xxviii^	106.96 (3)
F3^xviii^—Eu1—F3^xix^	147.22 (4)	Li2—F7—K2^xvii^	92.42 (8)
F5—Eu1—F1	139.47 (4)	K3^xvii^—F7—K2^xvii^	106.96 (3)
F2—Eu1—F1	72.63 (4)	K2^xxviii^—F7—K2^xvii^	81.97 (3)
F3^xviii^—Eu1—F1	75.29 (2)	Li2—F7—K2^xi^	78.37 (7)
F3^xix^—Eu1—F1	75.30 (2)	K3^xvii^—F7—K2^xi^	85.43 (3)
F5—Eu1—F4^xx^	76.34 (3)	K2^xxviii^—F7—K2^xi^	165.38 (4)
F2—Eu1—F4^xx^	77.25 (3)	K2^xvii^—F7—K2^xi^	87.055 (6)
F3^xviii^—Eu1—F4^xx^	140.49 (3)	Li2—F7—K2^xxix^	78.37 (7)
F3^xix^—Eu1—F4^xx^	72.04 (3)	K3^xvii^—F7—K2^xxix^	85.43 (3)
F1—Eu1—F4^xx^	133.97 (2)	K2^xxviii^—F7—K2^xxix^	87.055 (6)
F5—Eu1—F4	76.34 (3)	K2^xvii^—F7—K2^xxix^	165.38 (4)
F2—Eu1—F4	77.25 (3)	K2^xi^—F7—K2^xxix^	101.95 (4)
F3^xviii^—Eu1—F4	72.04 (3)	Li2—F8—Eu1^vi^	163.91 (12)
F3^xix^—Eu1—F4	140.49 (3)	Li2—F8—K1^i^	89.41 (9)
F1—Eu1—F4	133.97 (2)	Eu1^vi^—F8—K1^i^	102.73 (3)
F4^xx^—Eu1—F4	68.51 (4)	Li2—F8—K1^xxiii^	89.41 (9)
F5—Eu1—F8^xxi^	70.51 (4)	Eu1^vi^—F8—K1^xxiii^	102.73 (3)
F2—Eu1—F8^xxi^	141.59 (4)	K1^i^—F8—K1^xxiii^	81.17 (3)
F3^xviii^—Eu1—F8^xxi^	78.10 (2)	Li2—F8—K2^xxix^	76.74 (7)
F3^xix^—Eu1—F8^xxi^	78.10 (2)	Eu1^vi^—F8—K2^xxix^	93.11 (3)
F1—Eu1—F8^xxi^	68.96 (4)	K1^i^—F8—K2^xxix^	162.14 (4)
F4^xx^—Eu1—F8^xxi^	131.91 (2)	K1^xxiii^—F8—K2^xxix^	87.386 (10)
F4—Eu1—F8^xxi^	131.91 (2)	Li2—F8—K2^xi^	76.74 (7)
F6^xvii^—Li1—F1^xxii^	107.19 (16)	Eu1^vi^—F8—K2^xi^	93.11 (3)
F6^xvii^—Li1—F3^i^	107.75 (11)	K1^i^—F8—K2^xi^	87.386 (10)
F1^xxii^—Li1—F3^i^	105.42 (11)	K1^xxiii^—F8—K2^xi^	162.14 (4)
F6^xvii^—Li1—F3^xxiii^	107.75 (11)	K2^xxix^—F8—K2^xi^	100.09 (4)
F1^xxii^—Li1—F3^xxiii^	105.42 (11)		

Symmetry codes: (i) −*x*+1, −*y*+1, −*z*+1; (ii) *x*+1/2, *y*+1, −*z*+1/2; (iii) −*x*+1/2, −*y*+1, *z*−1/2; (iv) *x*, *y*+1, *z*; (v) −*x*+1/2, −*y*+1, *z*+1/2; (vi) *x*+1/2, *y*, −*z*+1/2; (vii) *x*−1/2, *y*+1, −*z*+3/2; (viii) −*x*+1/2, −*y*+2, *z*+1/2; (ix) −*x*+3/2, −*y*+1, *z*−1/2; (x) *x*−1/2, *y*, −*z*+3/2; (xi) −*x*+1, −*y*, −*z*+1; (xii) −*x*+1/2, −*y*, *z*+1/2; (xiii) −*x*+1/2, −*y*, *z*−1/2; (xiv) −*x*+1/2, *y*+1/2, *z*+1/2; (xv) *x*, *y*, *z*+1; (xvi) −*x*+1/2, *y*−1/2, *z*+1/2; (xvii) *x*+1/2, *y*, −*z*+3/2; (xviii) *x*, *y*−1, *z*; (xix) *x*, −*y*+3/2, *z*; (xx) *x*, −*y*+1/2, *z*; (xxi) *x*−1/2, *y*, −*z*+1/2; (xxii) *x*+1, *y*, *z*+1; (xxiii) −*x*+1, *y*−1/2, −*z*+1; (xxiv) *x*+1/2, −*y*+3/2, −*z*+3/2; (xxv) *x*+1/2, *y*−1, −*z*+3/2; (xxvi) −*x*+3/2, −*y*+1, *z*+1/2; (xxvii) −*x*+3/2, *y*−1/2, *z*+1/2; (xxviii) *x*+1/2, −*y*+1/2, −*z*+3/2; (xxix) −*x*+1, *y*+1/2, −*z*+1; (xxx) *x*−1, *y*, *z*−1; (xxxi) *x*−1/2, *y*−1, −*z*+1/2; (xxxii) *x*−1/2, −*y*+3/2, −*z*+1/2; (xxxiii) −*x*+1/2, *y*−1/2, *z*−1/2; (xxxiv) −*x*+1/2, −*y*+2, *z*−1/2; (xxxv) −*x*+1/2, *y*+1/2, *z*−1/2; (xxxvi) *x*, *y*, *z*−1.

## References

[bb1] Brandenburg, K. & Putz, H. (2006). *DIAMOND* Crystal Impact GbR, Bonn, Germany.

[bb2] Brown, I. D. (1992). *Acta Cryst.* B**48**, 553–572.

[bb3] Brown, I. D. (2002). *The Chemical Bond in Inorganic Chemistry - The Bond Valence Model* IUCr monographs on Crystallography 12. Oxford University Press.

[bb4] Gagor, A. (2009). Acta Cryst. E**65**, i81. 10.1107/S1600536809044043 PMC2971201 21578038

[bb5] Hong, H. Y.-P. & McCollum, B. C. (1979). *Mat. Res. Bull.* **14**, 137–142.

[bb6] Mattausch, H. J., Eger, R. & Simon, A. (1991). *Z. Anorg. Allg. Chem.* **597**, 145–150.

[bb7] Oxford Diffraction (2007). *CrysAlis CCD* and *CrysAlis RED* Oxford Diffraction Ltd, Abingdon, England.

[bb9] Ryba-Romanowski, W., Solarz, P., Gusowski, M. & Dominiak-Dzik, G. (2007). *Rad. Meas.* **42**, 798–802.

[bb10] Sheldrick, G. M. (2008). *Acta Cryst.* A**64**, 112–122. 10.1107/S0108767307043930 18156677

